# Supplementary material for: Measuring ligand-cell surface receptor affinities with axial line-scanning fluorescence correlation spectroscopy
Source: eLife. 2020 May 22;9:e55286. doi: 10.7554/eLife.55286 (PMC7289602; doi:10.7554/eLife.55286)
Supplement: Table 1—source data 1. [file elife-55286-table1-data1.docx]

**Table 1. Parameters obtained from the analysis of axial lsFCS data of DKK1 binding to LRP6-mCherry.**

| Cell line | HEK293T | | NCI-H1703 | | |
| --- | --- | --- | --- | --- | --- |
| Receptor gene insertion | Transient  transfection | | Transient transfection | Stable transfection | CRISPR/Cas9 genome editing |
| Ligand | DKK1-eGFP | DKK1-eGFP-SNAP | DKK1-eGFP | | |
| Receptor density (µm^‒2^) ^a^ | 370 ± 150 | 1080 ± 470 | 140 ± 80 | 45 ± 23 | 18 ± 9 |
| *K*_D_ (nM) | 0.49 ± 0.09 | 0.46 ± 0.09 | 0.22 ± 0.02 | 0.08 ± 0.01 | 0.09 ± 0.01 |
| *β* | 0.13 ± 0.01 | 0.15 ± 0.01 | 0.47 ± 0.01 | 0.44 ± 0.02 | 0.14 ± 0.01 |
| *D_G_* (µm^2^ s^‒1^) ^b^ | 0.44 ± 0.08 | 0.40 ± 0.07 | 0.45 ± 0.11 | 0.29 ± 0.06 | 0.33 ± 0.15 |
| *D_R_* (µm^2^ s^‒1^) ^b^ | 0.46 ± 0.09 | 0.45 ± 0.11 | 0.42 ± 0.13 | 0.27 ± 0.05 | 0.33 ± 0.06 |
| $\left\langle\gamma_{G} \right\rangle$ ^c^ | 1.32 ± 0.11 | 1.25 ± 0.24 | 1.36 ± 0.21 | 1.62 ± 0.25 | 1.54 ± 0.23 |
| $\left\langle\gamma_{R} \right\rangle$ ^c^ | 1.21 ± 0.08 | 1.26 ± 0.13 | 1.33 ± 0.16 | 1.54 ± 0.25 | 1.42 ± 0.29 |

^a^ Receptor densities are given as median ± half the range covered by the second and third quartile of the distribution. Individual data points were calculated from receptor autocorrelation amplitudes, *G*_R_(0), and the observation area (0.20 µm^2^).

^b^ Diffusion coefficients of receptor-bound ligands and receptors were calculated from the translational diffusion times by using *D* = *ω*_0_^2^/4*τ*.

^c^ The photobleaching parameters 〈*γ*〉 are given as the median value ± half the range covered by the second and third quartile of the distribution compiled from all scans.
