# Supplementary material for: Measuring ligand-cell surface receptor affinities with axial line-scanning fluorescence correlation spectroscopy
Source: eLife. 2020 May 22;9:e55286. doi: 10.7554/eLife.55286 (PMC7289602; doi:10.7554/eLife.55286)
Supplement: Table 2—source data 1. [file elife-55286-table2-data1.docx]

**Table 2. Parameters obtained from the analysis of axial lsFCS data on NCI-1703 cells.**

| Receptor | LRP6-mCherry | LRP6-tdTomato | LRP6-tdTomato | xKremen2-mCherry | xKremen2-mCherry, LRP6 |
| --- | --- | --- | --- | --- | --- |
| Receptor gene insertion | Stable transfection | Transient transfection | CRISPR/Cas9 genome editing | Transient transfection | Transient  transfection |
| Ligand | DKK2-eGFP | DKK1-eGFP | DKK1-eGFP | DKK1-eGFP | DKK1-eGFP |
| Receptor density (µm^‒2^) ^a^ | 32 ± 16 | 106 ± 52 | 21 ± 15 | 44 ± 33 | 41 ± 30 |
| *K*_D_ (nM) | 0.26 ± 0.04 | 0.22 ± 0.03 | 0.10 ± 0.02 | 10.3 ± 2.1 | 0.54 ± 0.06 |
| *β* | 0.43 ± 0.02 | 0.51 ± 0.02 | 0.26 ± 0.01 | 0.45 ± 0.02 | 0.66 ± 0.03 |
| *D_G_* (µm^2^ s^‒1^) ^b^ | 0.27 ± 0.08 | 0.35 ± 0.19 | 0.27 ± 0.19 | 0.25 ± 0.15 | 0.32 ± 0.18 |
| *D_R_* (µm^2^ s^‒1^) ^b^ | 0.30 ± 0.07 | 0.44 ± 0.34 | 0.39 ± 0.19 | 0.19 ± 0.08 | 0.27 ± 0.09 |
| $\left\langle\gamma_{G} \right\rangle$ ^c^ | 1.22 ± 0.17 | 1.31 ± 0.15 | 1.47 ± 0.25 | 1.36 ± 0.22 | 1.30 ± 0.22 |
| $\left\langle\gamma_{R} \right\rangle$ ^c^ | 1.41 ± 0.19 | 1.41 ± 0.16 | 1.66 ± 0.39 | 1.42 ± 0.29 | 1.35 ± 0.16 |

^a^ Receptor densities were calculated from the amplitudes of the receptor autocorrelation functions, *G*_R_(0), and the observation area (0.20 µm^2^).

^b^ Diffusion coefficients of receptor-bound ligands and receptors were calculated from the translational diffusion times by using *D* = *ω*_0_^2^/4*τ*.

^c^ The photobleaching parameters 〈*γ*〉 are given as the median of the distribution from all scans; the errors denote half the width of the second and third quartile of the distribution.
